# Ornithine Decarboxylase Antizyme Induces Hypomethylation of Genome DNA and Histone H3 Lysine 9 Dimethylation (H3K9me2) in Human Oral Cancer Cell Line

**DOI:** 10.1371/journal.pone.0012554

**Published:** 2010-09-03

**Authors:** Daisuke Yamamoto, Kaori Shima, Kou Matsuo, Takashi Nishioka, Chang Yan Chen, Guo-fu Hu, Akira Sasaki, Takanori Tsuji

**Affiliations:** 1 Department of Radiation Oncology, Beth Israel Deaconess Medical Center, Harvard Medical School, Boston, Massachusetts, United States of America; 2 Department of Oral and Maxillofacial Surgery, Okayama University Graduate School, Okayama, Japan; 3 Center for Molecular Oncologic Pathology, Dana-Farber Cancer Institute, Harvard Medical School, Boston, Massachusetts, United States of America; 4 Division of Oral Pathology, Department of Biosciences, Kyushu Dental College, Kitakyushu, Japan; 5 Department of Pathology, Harvard Medical School, Boston, Massachusetts, United States of America; New England Biolabs, Inc, United States of America

## Abstract

**Background:**

Methylation of CpG islands of genome DNA and lysine residues of histone H3 and H4 tails regulates gene transcription. Inhibition of polyamine synthesis by ornithine decarboxylase antizyme-1 (OAZ) in human oral cancer cell line resulted in accumulation of decarboxylated *S-*adenosylmethionine (dcSAM), which acts as a competitive inhibitor of methylation reactions. We anticipated that accumulation of dcSAM impaired methylation reactions and resulted in hypomethylation of genome DNA and histone tails.

**Methodology/Principal Findings:**

Global methylation state of genome DNA and lysine residues of histone H3 and H4 tails were assayed by Methylation by Isoschizomers (MIAMI) method and western blotting, respectively, in the presence or absence of OAZ expression. Ectopic expression of OAZ mediated hypomethylation of CpG islands of genome DNA and histone H3 lysine 9 dimethylation (H3K9me2). Protein level of DNA methyltransferase 3B (DNMT3B) and histone H3K9me specific methyltransferase G9a were down-regulated in OAZ transfectant.

**Conclusions/Significance:**

OAZ induced hypomethylation of CpG islands of global genome DNA and H3K9me2 by down-regulating DNMT3B and G9a protein level. Hypomethylation of CpG islands of genome DNA and histone H3K9me2 is a potent mechanism of induction of the genes related to tumor suppression and DNA double strand break repair.

## Introduction

**Figure 1:**
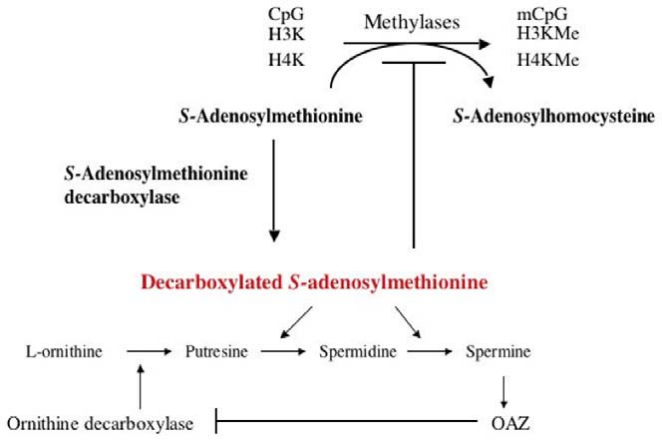
Metabolic pathway coupling between polyamine synthesis and methylation reactions. ODC is a key enzyme of polyamine synthesis by catalyzing from L-ornithine to putrescine. OAZ is an inhibitory molecule of ODC activity by mediating degradation of ODC protein. SAM serves as a methyl donor in methylation reactions as well as precursor of dcSAM production. dcSAM is an aminopropyl donor of polyamine biosynthesis but it also acts as a competitive inhibitor of SAM virtually in all methylation reactions. Inhibition of ODC activity leads to polyamine depletion and dcSAM accumulation, which inhibits methylation reactions.

Ornithine decarboxylase (ODC; EC4.1.1.17) is the first and rate-limiting key enzyme for polyamine biosynthesis by catalyzing from L-ornithine to putrescine [1] and is implicated in cell proliferation and differentiation [2]. Ornithine decarboxylase antizyme subfamily consists of three related antizyme members and ornithine decarboxylase antizyme-1 (OAZ) was first discovered and identified as an ornithine decarboxylase (ODC) inhibitory molecule by stimulating degradation of ODC protein [3], [4]. OAZ is known to bind various proteins and mediate degradation or stabilization of the target proteins. These proteins include ODC [5], antizyme inhibitor [6], cyclin D1 [7], [8] and HPV16 E2 [9]. As shown in Fig. 1, inhibition of ODC activity and the subsequent polyamine synthesis by OAZ [10] or chemical compound, α-difluoromethylornithine (DFMO) [5] resulted in accumulation of dcSAM. dcSAM serves as an aminopropyl donor for polyamine synthesis but it acts as a competitive inhibitor of S-adenosylmethionine, virtually in all methylation reactions including DNA, RNA, lipid and protein [5], [11]. Ectopic expression of OAZ in hamster oral cancer cells induced global hypomethylation, and transactivated the genes related to tumor suppression and epithelial differentiation [10]. Subsequently, we profiled the genes induced or up-regulated in human oral cancer cells by OAZ, and found induction of genes related to DNA double strand break repair by non-homologous end-joining mechanism [10].

DNA methylation of CpG islands and histone modification, particularly acetylation and methylation of lysine residues of histone tails are tightly correlated to regulate gene expression. Histone acetylation is generally correlated with transcriptional activation but histone methylation regulates transcriptional activation or repression depending on the site of lysine methylation [12]. Histone lysine methylation occurs on six lysine residues of the tails from histones H3 (K4, K9, K27, K36, K79) and H4 (K20) [13], [14]. Each of these lysines can be mono-, di- or trimethylated [13]. Methylation of H3K4, H3K36 and H3K79 are mainly correlated with transcriptional activation whereas methylation of H3K9, H3K27 and H4K20 occurs mainly in association with transcriptional silencing [15], [16]. The interdependent relationship between DNA methylation and histone methylation is one of the interest topics in gene regulation. H3K9 methylation and DNA methylation are tightly associated in heterochromatin and transcriptionally repressed euchromatic regions. Growing evidences unveiled that DNA cytosine methylation co-exists with repressive H3K9 methylation because DNA methyltransferases, methyl-CpG binding protein (MECP2), and heterochromatin protein1 (HP1) recruit H3K9 specific methyltransferase to methylate histone lysine residues [17]–[19]. This mechanism is reversed in some systems [20]. H3K9 methylation is a prerequisite for DNA methylation [21]–[24]. We hypothesize that accumulation of dcSAM by ectopic OAZ expression leads to hypomethylation of genome DNA and histone tails and the hypomethylation of genome DNA and histone tails up-regulates or transactivates the genes involved in DNA double strand break repair. The genomic data serve as a foundation to understand the interrelationship between transcription factor and chromatin based pathways. In the present study, we screened and verified OAZ mediated global hypomethylation of genome DNA and histone H3 and H4 tails.

## Results

### ODC activity

**Figure 2:**
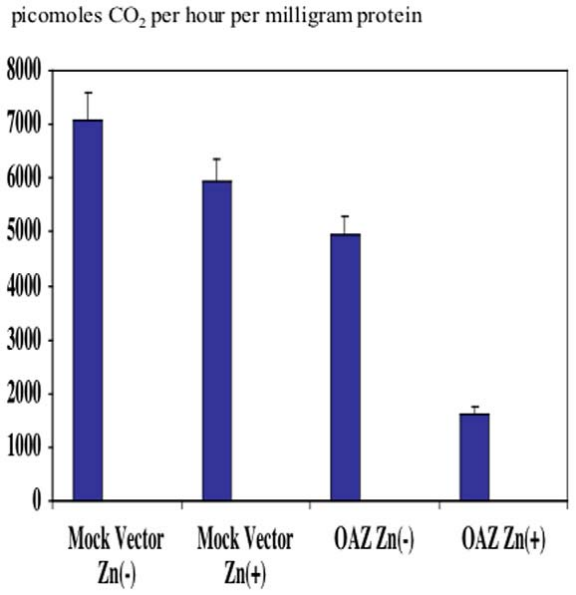
ODC enzymatic activity assay in mock vector and OAZ transfectants. ODC activity (picomoles CO_2_ per hour per milligram protein) of the parental UM1, the mock vector transfectant and OAZ transfectant with and without ZnSO_4_ treatment was measured. Triplicate quantification was performed. Corrected ODC activity values were obtained by subtracting the value for blank. ODC activity of OAZ transfectant without ZnSO_4_ treatment was suppressed because trace amount of ZnSO_4_ in culture medium caused leakage of OAZ gene expression.

OAZ protein promotes degradation of ornithine decarboxylase (ODC) protein and reduction of ODC enzyme activity. To examine whether OAZ protein transcribed and translated from the OAZ transfectant (UM1-pMT/CB6^+^HuFSAZ-wt) is a biologically active protein, ODC enzyme activity assay was performed as previously described [10]. The ODC activity in the OAZ transfectant with ZnSO_4_ treatment exhibited reduced ODC enzyme activity (1629.0±61.7/picomoles CO_2_ per hour per milligram protein) by 72.6% comparing with that of the mock vector transfectant (UM-1pMT/CB6^+^) treated with ZnSO_4_ (5930.0±110.7/picomoles CO_2_ per hour per milligram protein) (p<0.05). The ODC activity in the OAZ transfectant without ZnSO_4_ treatment decreased (4944.1±414.6/picomoles CO_2_ per hour per milligram protein) by 30.3% comparing with that of the mock vector transfectant without ZnSO_4_ treatment (7084.7±284.1/picomoles CO_2_ per hour per milligram protein) (Fig. 2). This is probably due to the leakage of gene expression induced by trace amount of Zn^2+^ supplemented in the culture medium. The reduction of ODC activity in the OAZ transfectant confirmed that OAZ protein induced by ZnSO_4_ treatment was biologically functional protein.

### Intracellular level of polyamines and metabolites

**Figure 3:**
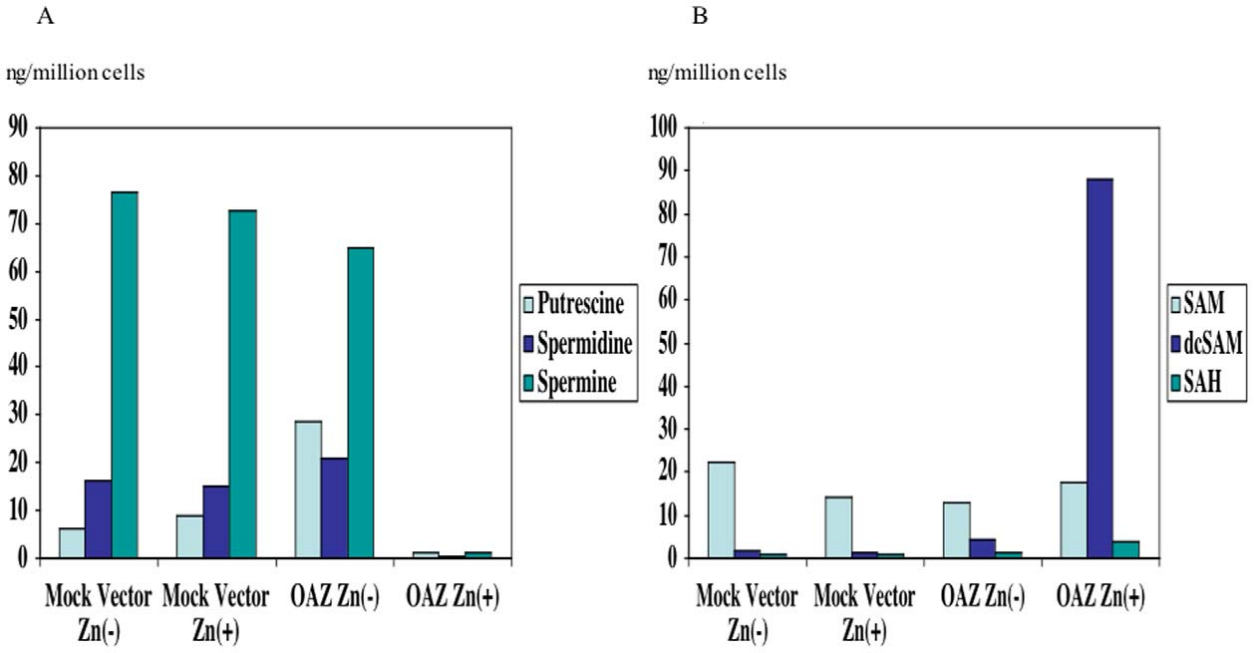
Intracellular level of polyamines, L-ornithine, SAM, dcSAM, SAH. Polyamine pool was depleted (A) but dcSAM and SAH, which were potential inhibitors of methylation reactions, increased (B) in the OAZ transfectant treated with ZnSO_4_. Cellular level of SAM was constant through the experiments (B).

We anticipated that reduction of ODC enzymatic activity led to the depletion of polyamine pool and the subsequent alteration of intracellular level of polyamine metabolites. We quantified intracellular ODC, polyamines and metabolites level by HPLC analysis as previously described [5]. Ectopic expression of OAZ down-regulated ODC protein level from 51ng/10^6^ cells to 8.4 ng/10^6^ cells. As anticipated, the polyamine levels dramatically decreased as result of suppression of ODC activity. Putrescine level decreased from 9 ng/10^6^ cells to 1.3 ng/10^6^ cells. Spermidine level decreased from 15.1 ng/10^6^ cells to 0.2 ng/10^6^ cells. Spermine level decreased from 72.8 ng/10^6^ cells to 1.0 ng/10^6^ cells (Fig. 3A). S-adenosylmethionine (SAM), which is a methyl donor for all methylation reactions, was quite constant through the experiments. The intracellular amount of SAM was 17.8 ng/10^6^ cells in the OAZ transfectant and 14.3 ng/10^6^ cells in the mock vector transfectant. The intracellular level of dcSAM in the control mock vector transfectant was 1.22 ng/10^6^ cells but it drastically increased to 87.9 ng/10^6^ cells in the OAZ transfectant. The intracellular level of S-adenosylhomocystein (SAH) also increased from 0.66 ng/10^6^ cells to 3.76 ng/10^6^ cells (Fig. 3B).

### Methylation state of genome DNA

**Figure 4:**
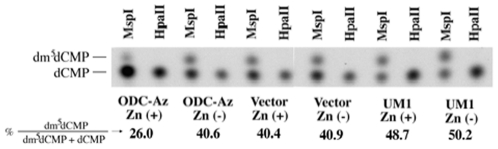
Quantification of global DNA methylation in the parental UM1 cells, mock vector and OAZ transfectants. Global methylation level of 5′-methyl-CMP (dm5′dCMP) and 5′dCMP in genome DNA of the parental UM1 cells, mock vector and OAZ transfectant was measured by scintillation counting. ^32^P-labeled dm^5^CMP and CMP were produced by cleavage of the respective genome DNAs with the restriction enzymes *Msp*I and its methylation sensitive isoschizomer *Hpa*II and they were separated on a thin layer chromatography.

As we have already published, ectopic expression of OAZ in the hamster malignant oral keratinocytes induced global hypomethylation of CCGG sites of the genome DNA [10]. In the present study, we examined whether the same hypomethylation was induced by the human OAZ in human oral cancer cell line. The level of methylated cytosines in CCGG sites was quantified by measuring the radioactivity within the two major spots, 5′[^32^P]dCMP and 5′[^32^P]dm^5^CMP by liquid scintillation counting. The result of the quantification is shown in Fig. 4 as a percentage of dm^5^CMP. The OAZ transfectant with and without ZnSO_4_ treatment exhibited that 26.0% and 40.6% of CCGG sequences were methylated, respectively. The parental UM1 cells with and without ZnSO_4_ treatment, and the mock vector transfectant with and without ZnSO_4_ treatment exhibited 48.7% and 50.2%, and 40.4% and 40.9% of the internal cytosine was methylated, respectively. This result indicates ectopic expression of OAZ is associated with genome-wide DNA demethylation in human oral cancer cell line. Genomic DNA of the OAZ transfectant is about 60% less methylated than that of the mock vector transfectant.

### Methylation state of histone H3 and H4 tails

**Figure 5:**
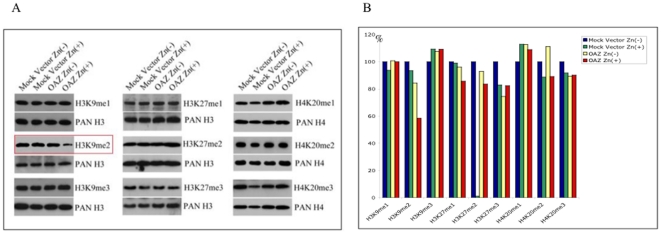
Western immunoblot analysis of global methylated lysine residues of the histone H3 and H4 in mock vector and OAZ transfectants. Significant hypomethylation of H3K9me2 was shown in the OAZ transfectant treated with ZnSO_4_. Methylation status of other lysine residues of histone H3 and H4 tails was not altered. Histone H3 and H4 proteins (17 kDa and 10 kDa, respectively) was also blotted on the same membrane after stripping to monitor equal amount of protein loading in each lane. We show only H3K9, H3K27 and H4K20 results because methylation of these histone tails works as transcriptional repressor (A). The value denote the relative intensity of methylated histone protein bands of the OAZ tranfectants to that of the mock vector transfectants after being normalized to the PAN H3 and H4 bands. Intensity of the control (mock vector transfectant without ZnSO_4_ treatment) was considered as 100 and other signals were expressed as relative value (B).

Methylation state of the lysine residues of histone H3 and H4 tails links to formation of transactive euchromatin or transrepressive heterochromatin depending on methylation site. Depletion of polyamine pool by OAZ led to accumulation of dcSAM, which acts as competitive inhibitor of SAM in methylation reactions. We anticipated accumulation of dcSAM mediated global hypomethylation of lysine residues of histone H3 and H4 tails. As the first screening approach to explore a potential connection between accumulation of dcSAM and alteration of global histone methylation state, we analyzed the global changes in the methylation status of histne H3 and H4 by western blotting. Sixteen antibodies against mono-, di- and tri-methylation of specific lysine residues of histone H3 and H4 were used. We could observe a significant decrease in global level of histone H3 Lys-9 dimethylation (H3K9me2), H3K27me1, H3K27me2, an H3K27me3 by 41.6, 14.3, 16.4 and 17.5% respectively in the OAZ transfectant (Fig. 5A and B). The signal intensity of histone H3 was constant among all samples loaded. In contrast, other twelve antibodies for histones H3 (H3K4me1, H3K4me2, H3K4me3, H3K9me1, H3K9me3, H3K36me1, H3K36me2, H3K36me3, H3K79me2) and H4 (H4K20me1, H4K20me2, H4K20me3) did not exhibit any significant difference between the OAZ transfectant and the mock vector transfectant (Fig. 5A and B). Di-methylation of histone H3K9 is a mark of constitutive heterochromatin and gene repression. Hypomethylation of H3K9me2 represents a change of chromatin state from heterochromatin to euchromatin, which also means transcriptional activity of genes changes from repressive state to active state. This demethylation of H3K9me2 by OAZ speculates activation of the genes related to differentiation [10] and DNA double strand break repair [25].

### Expression level of DNMTs and G9a

**Figure 6:**
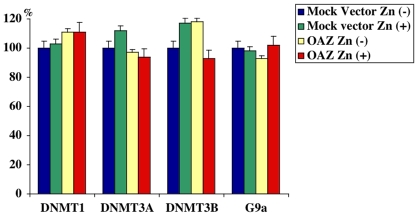
Expression level analysis of DNMT1, 3A, 3B and G9a mRNA in mock vector and OAZ transfectants by qRT-PCR. Expression level of these mRNA was not altered by OAZ. The PCR result was normalized using the -beta-actin signal. Signal of the control (mock vector transfectant without ZnSO_4_ treatment) was considered as 100 and other signals were expressed as relative value.

**Figure 7:**
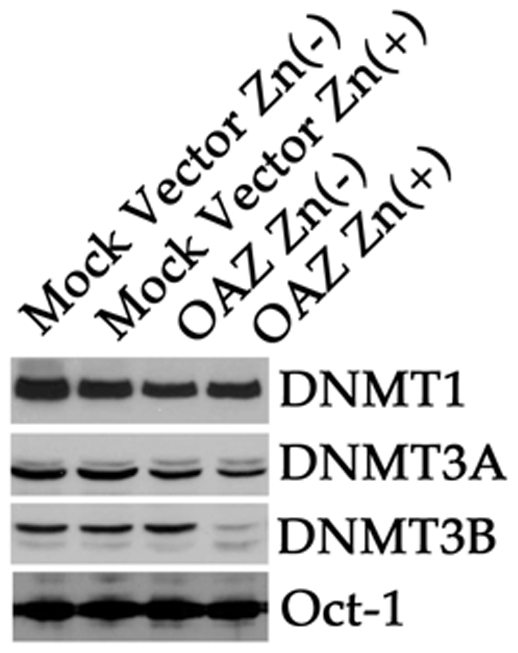
Western immunoblot analysis of DNMTs proteins in mock vector and OAZ transfectants. Nuclear extracts were immunoblotted with anti-DNMTs antibodies. Protein level of DNMT1 (183 kDa) and DNMT3A (101 kDa) was constant but protein level of DNMT3B (110 kDa) was significantly decreased in the OAZ transfectant treated with ZnSO_4_. The fain bands appeared in the DNMT3A and DNMT3B blots are isoforms of DNMT3A and 3B. Oct-1 (89 kDa) was used to monitor equal amount of protein loading in each lane.

**Figure 8:**
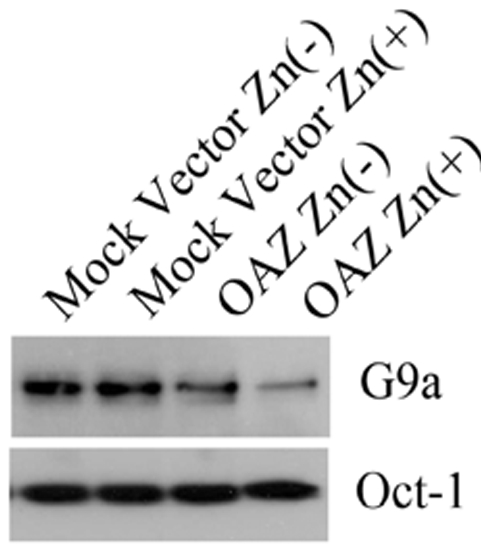
Western immunoblot analysis of G9a protein in mock vector and OAZ transfectants. Nuclear extracts were immunoblotted with anti-G9a antibody. Expression of G9a protein (140 kDa) was decreased in the OAZ transfectant treated with ZnSO_4_.

We first examined whether OAZ altered the mRNA expression level of DNMTs and G9a by qRT-PCR. Conventional RT-PCR was performed prior to qRT-PCR to confirm that the primer set was able to amplify single amplicon. We confirmed these PCR primers could amplify crispy single band after 35 cycle PCR reaction (data not shown). The subsequent qRT-PCR results revealed that OAZ expression or ZnSO_4_ treatment did not affect the expression level of DNMT1, 3A, 3B and G9a mRNA (p<0.01) (Fig. 6). OAZ mediated global hypomethylation of genome DNA and histone H3K9me2. DNMTs 1, 3A, 3B and G9a/GLP histone methyltrasferase complex are involved in methylation of genome DNA and histone H3K9me2, respectively. We hypothesized that hypomethylation of histone tails was one of pleiotropic targets of dcSAM-mediated hypomethylation, and all of the methylated lysines of histone tails were hypomethylated but in fact only H3K9me2 was hypomethylated. This unexpected result prompted us to examine the protein level of DNMTs and G9a. Methylation of the mammalian H3K9 is catalyzed by G9a/GLP histone methyltransferase complex. Western blot analyses revealed the protein level of DNMT3B (Fig. 7) and G9a (Fig. 8) was significantly down-regulated but the protein level of DNMT1 and 3A was not altered in the OAZ transfectant. This outcome indicates that global hypomethylation of genome DNA and histone tails is caused by not only by a pleiotropic effect of dcSAM but specific manner works to hypomethylate genome DNA and histone tails.

### DNMT enzyme activity

**Figure 9:**
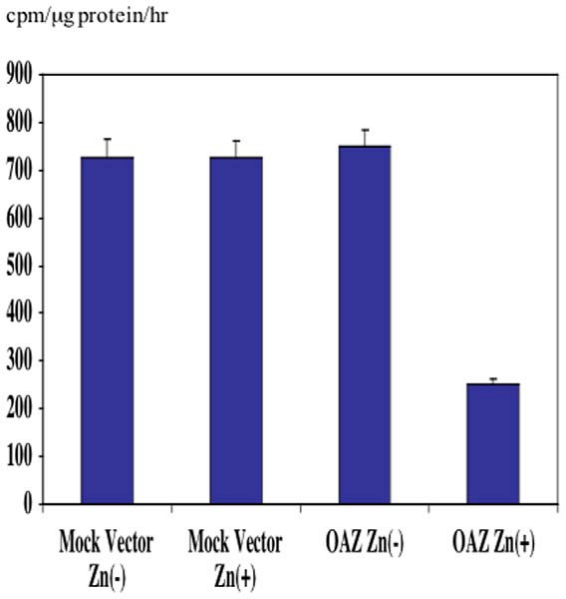
DNMT enzymatic activity assay in mock vector and OAZ transfectants. Total DNMT activity assay was performed. DNMT activity was decreased by ∼65% in the OAZ transfectant treated with ZnSO_4_. This result is correlated with the western blot result of DNMT3B.

We used poly(dI-dC)poly(dI-dC) as a substrate for the DNMT enzyme activity assay. DNMT enzyme activity was determined by measuring incorporation of label into poly(dI-dC)poly(dI-dC) substrate. Methyl poly(dI-dC)poly(dI-dC) production was suppressed by ∼65% in the OAZ transfectant with ZnSO_4_ treatment comparing with those of the control transfectant and the OAZ transfectant without ZnSO_4_ treatment (P<0.01) (Fig. 9). This result revealed that decreased DNMT3B protein suppressed the part of total DNMT activity and this decreased DNMT activity is correlated with down-regulation of DNMT3B protein by OAZ.

## Discussion

We have reported that ectopic expression of OAZ in hamster oral cancer cells resulted in global hypomethylation of genome DNA [10] and induced or up-regulated expression of the genes related to DNA double strand break repair in human oral cancer cells [25]. Aberrant methylation of CpG islands within the promoter region inactivates gene transcription [26]–[28]. Covalent modifications of histone tails also play important roles in transcription, DNA replication, DNA break repair and chromatin condensation in mitosis [29]. Among these histone modifications, methylation and acetylation of histone tails are involved in regulation of gene transcription [30], [31]. In the present study, we examined the global methylation state of genome DNA and lysine residues from histone tails in human oral cancer cells in the absence or presence of OAZ expression. Inhibition of polyamine synthesis by OAZ accumulated abnormally high level of dcSAM, which acts as a competitive inhibitor of *S-*adenosylmethionine virtually in all methylation reactions. We predicted accumulation of dcSAM induced hypomethylation of cystine residues of CpG islands and altered methylation marks of lysine residues of histone tails. Cytosine residues of CCGG sites in genome DNA were globally hypomethylated as anticipated but only H3K9me2 was hypomethylated among the methylable lysine residues of histone tails. These results prompted us to examine the protein level of DNA methyltransferases (DNMTs) and H3K9me2-specific methyltransferase G9a. Our western blot results exhibited protein level of DNMT3B and G9a was down-regulated in the OAZ transfectants. DNA methylation patterns are established and maintained by the coordination of DNA methyltransferases, DNMT1, DNMT3A and DNMT3B. DNMT1 pursues maintenance of methylation pattern following DNA replication, whereas DNMT3A and DNMT3B are mainly responsible for de novo methylation [32] and maintenance of methylation in certain regions of the genome DNA during embryogenesis and development [33]. DNMT3B depletion in human cancer cell lines reactivated methylation-silenced gene expression but did not induce global or juxtacentromeric satellite demethylation [34]. Depletion of DNMT3B by OAZ might promote the sequence-specific DNA demethylation and induced or up-regulated the expression of genes related to DNA break repair but accumulation of dcSAM also contributed to random, global DNA demethylation.

G9a histone methyltransferase, which uniquely methylates histone H3K9, is also a major player of gene silencing [35] and essential for early embryogenesis to regulate developmental gene expression [36]. Methylation of H3K9me2 mediated by G9a alters chromatin structure from relaxed euchromatin to compact heterochromatin and represses a number of gene expression [36]–[38]. Methylation of H3K9 has been known to associate with hypermethylation of promoter CpG islands in cancer cells [39], [40]. DNA methylation and H3K9 methylation tightly cooperate to regulate gene expression. H3K9 methylation is a prerequisite for DNA methylation [21]–[23] and H3K9 methylation is required for DNA methylation [22], [23]. G9a mediated DNA methylation does not require its catalytic activity [41], suggesting that it may have additional functions in directing DNA methylation, such as the recruitment of DNMTs [42]. G9a protein binds to DNMT1 and leads to enhanced DNA and histone methylation [43]. G9a protein also recruits and binds to DNMT3A and DNMT3B proteins through its ankyrin (ANK) domain [44], and locates them to the sites of DNA replication. H3K9me2 plays equally important roles in gene silencing in euchromatin and subsequent de novo DNA methylation of embryonic and germ line genes during normal development [41], and is necessary for the maintenance of DNA methylation at endogeneous retrotransposons, imprinted loci, and other genes in differentiated cells [45]. Li et al and Deng et al reported inhibition of global genome DNA methylation with 5-Aza-2′-deoxycytidine (5-Aza-CdR) decreased the protein expression of DNMT1 and DNMT3B [46], [47]. Wozniak et al reported that 5-Aza-CdR treatment hypomethylated global H3K9me2 in human breast cancer cells by down-regulating G9a protein level [48]. These data suggest that DNA demethylation may be a signal that directs the demethylation of H3K9 during DNA replication.

It is an odd result that accumulation of dcSAM did not alter methylation state of lysine residues of histone tails other than H3K9me2 because inhibition of methylation by dcSAM seems a pleiotropic effect. Transcription factors also have pleiotropic effect but their activity is tightly regulated by cis- and trans-acting elements to regulate gene expression in tissue-, developmental stage-, and disease-specific manner. We believe methylation inhibition by dcSAM is not pleiotropic manner but it is also regulated by additional cis- and tras-acting elements to target specific sites of the genes and histone tails. The precise molecular mechanism underlying the down-regulation of DNMT3B and G9a proteins by OAZ remains elusive. We propose a potent molecular mechanism that OAZ protein directly or indirectly bind to DNMT3B and/or G9a proteins and promotes their degradation. OAZ protein has been known to bind various proteins and promotes their degradation [7]–[9], [49]–[51]. The current our result underscores the complex relationship of histone methylation and susceptibility to DNA methylation. These evidences suggest that expression of genes related to DNA repair [25] may be activated by demethylation of CpG islands and H3K9me2 within the regulatory regions.

## Materials and Methods

### Cell culture

Human oral cancer cell line, UM1 [52] and the zinc-inducible OAZ transfectant (pMT/CB6^+^HuFSAZ-wt) and the mock vector transfectant (UM1-pMT/CB6^+^) were cultured as previously described [25]. The expression of OAZ gene from the pMT/CB6^+^HuFSAZ-wt was induced by 100 µM ZnSO_4_ treatment and the protein samples were harvested after one week ZnSO_4_ treatment.

### ODC activity assay

To check whether OAZ protein translated from the OAZ transfectant was biologically active protein in the U1 cells, ODC enzymatic activity was assayed as previously described [10]. Briefly, the ODC activity was quantified the release of [^14^C] CO_2_ during ODC-catalyzed conversion of L-ornithine to putrescine. The reaction mixture contained 50 µl of cell lysate prepared from the OAZ transfectant and the mock vector transfectant with and without ZnSO_4_ treatment, 75 µl of 100 mM glycyl-glycine (pH 7.2), 0.2 mM pyridoxal phosphate, 4 mM dithiothreitol, 0.4 mM L-ornithine, and 0.25 µCi [^14^C] l-ornithine. Reactions were carried out at 37°C for 2hr and terminated by heating at 85°C for 5 min. [^14^C]CO_2_ was trapped with Whatman 3-mm paper filter spotted wit 10 µl of 10% w/v KOH and quantified by scintillation counter. Triplicate assays were performed. Control sample was the blank containing lysis buffer in place of the supernatant. Corrected ODC activity values were obtained by subtracting values for blank and stated as picomoles CO_2_ per hour per milligram protein. The statistic analysis was performed by One factor ANOVA analysis.

### HPLC analysis

Intracellular amount of ODC, polyamines, SAM, dcSAM and SAH (S-adenosylhomecysteine) was quantified by reversed-phase HPLC analysis. The whole cell lysates from the OAZ transfectant and the mock vector transfectat with and without ZnSO_4_ treatment were prepared in 0.2 M perchloric acid. The cell lysates were centrifuged at 13,000 X g for 10 min and the supernatants were subjected to reversed-phase HPLC analysis on a Supercosil LC-18-DB column (4.6×250 mm, 5 µm pore size) equilibrated with 0.2% triethylamine-phosphoric acid (pH 4.0). Elution was achieved with 20 min linear gradient from 0–10% acetonitrile. The aliquots were subjected to chromatographic separation. The standards for ODC, polyamines, SAM and SAH were purchased from Sigma-Aldrich. The standard for dcSAM was a kind gift from Dr. Keijiro Samejima of Josai University in Japan.

### Western blot analysis of methyl-histone tails

Methylation status of each histone tails was verified by western blotting. The protin samples for western blot analyses of histone tails were prepared by directly adding 500 µl of Laemmli's SDS-sample buffer into 100mm culture plate and the cells were harvested by scrapping with a rubber policeman. The harvested cells were boiled for 15 minutes in the sample buffer and centrifuged at 16,000 X g for 30 minutes. The protein amount was estimated by the cell number of the replicated culture. The extracted proteins equivalent to 1×10^5^ – 5×10^5^ cells were loaded and separated in 15% SDS-PAGE gel, transferred to a PVDF membrane, blocked with 5% milk in TBS-T buffer and blotted with each antibody at recommended dilution by manufactures. The signals were developed with Pierce's SuperSignal® West Pico Chemiluminescent Substrate and autographed on film. To eliminate potential artifact caused by zinc, we repeated the same experiments using the pCI-neo-hOAZ tarsnfectants (pCI-neo-hOAZ-UM1). The antibodies used in this study were purchased from Upstate Biotechnology. The antibodies and their catalog numbers are; pan-histone H3 (07-690), pan-histone H4 (05-858), H3K4me1 (07-436), H3K4me2 (07-030), H3K4me3 (07-473), H3K9me1 (07-450), H3K9me2 (07-441), H3K9me3 (07-442), H3K27me1 (07-448), H3K27me2 (07-452), H3K27me3 (05-851), H3K36me1 (05-800), H3K36me2 (07-369), H3K36me3 (05-801), H3K79me2 (05-835), H4K20me1 (05-735), H4K20me2 (07-367), H4K20me3 (07-463). The secondary antibodies used were HRP-labeled goat anti-mouse IgG (PerkinElmer, NEF822001EA) and HRP-labeled goat anti-rabbit IgG (PerkinElmer, NEF812001EA). The intensity of each band was measured and analyzed using ImageJ software provided by NIH (http://rsb.info.nih.gov/ij/).

### Quantitative real-time PCR assay of DNMTs and G9a

Expression level of DNMTs and G9a mRNAs was quantified by quantitative real-time PCR (qRT-PCR). Total RNA was isolated from the OAZ and the mock vector transfectants using TRIzol® reagent (Invitrogen) according to the manufacture's protocol. The PCR primers were designed using MacVector version 7.2 software based on the cDNA sequences that were downloaded from the NCBI database. The qRT-PCR reactions were performed using a LightCycler with LightCycler FastStart DNA Master SYBR Green I kit. Amplification of sample cDNA was monitored with the fluorescent DNA binding dye SYBR Green in combination with an ABI 5700 sequence detection system. β-actin was used as an endogenous control for normalization. The PCR primer sequences, optimal annealing temperatures, and amplicon sizes are listed in Table 1. The statistic analysis was performed by One factor ANOVA analysis.

**Table 1:** List of PCR primers for qRT-PCR.

Genes	Primer sequences	Annealing temperature (oC)	Amplicon (nt)
Beta-actin	F: 5′-TGAAGGTGACAGTCGGTTG-3′	56	146
	R: 5′-GGCTTTTAGGATGGCAAGGGAC-3′		
DNMT1	F: 5′-AGGGAAAAGGGAAGGGCAAG-3′	57	103
	R: 5′-CCAGAAAACACATCCAGGGTCC-3′		
DNMT3A	F: 5′-CCATAAAGCAGGGCAAAGACC-3′	52	105
	R: 5′-AGTGGACTGGGAAACCAAATACC-3′		
DNMT3B	F: 5′-TTTTCCCCCACAAACCCAAG-3′	56	105
	R: 5′-TAGAACTCAGCACACCCTTCCTG-3′		
G9a	F: 5′-CAAGGATGGAGAGGTGTACTG-3′	52	196
	R: 5′-GTCGCCATAGTCAAACCCTAG-3′		

### Western blot analysis of DNMTs and G9a proteins

The nuclear extract was prepared from the OAZ and the mock vector transfectants using NE-PER® Nuclear and Cytoplasmic Extraction Reagents (Thermo Scientific) according to the manufacture's protocol. Fifty µg of nuclear extract of each sample was loaded and separated in 5% SDS-PAGE gel and the protein was transferred onto a PVDF membrane. The subsequent western blotting was performed as described above. The antibodies used for this study were a rabbit polyclonal anti-human DNMT1 antibody (abcam, ab19905, 1∶500), a rabbit-polyclonal anti-mouse DNMT3A antibody (abcam, ab23565, 1∶200), a rabbit polyclonal anti-mouse DNMT3B antibody (abcam, ab16049, 1∶300) and a rabbit polyclonal anti-human G9a antibody (Upstate Biotechnology, 07-551,1∶1,000). The equal amount of sample loading was monitored and confirmed by Oct-1signal using a rabbit polyclonal anti-human Oct-1 antibody (Santa Cruz Biotechnology, Inc., sc-232, 1∶1,000).

### DNMT enzyme activity assay

DNMT enzyme activity was determined by the method of Belinsky et al with minor modifications [53]. Cell lysate was prepared in lysis buffer by freezing-thawing three times and centrifuged to remove cell debris. Cell lysate containing 20 µg protein (125 µl) was mixed with 125 µl reaction mixture (20 mM Tris HCl, pH = 7.4, 25% glycerol, 5 mM EDTA, 1 mM DTT, 5 µCi [methyl-3H]-S-adenosyl-L-methionine, 4 µg poly [dI-dC]poly[dI-dC], 25 µg BSA) and incubated for 2 h at 37°C. Reactions were stopped and the DNA was extracted with Phenol:chrolform extraction method. The aqueous solution was supplemented with 0.1N NaOH and incubated for 2 hr at 50°C. The reaction mixture was neutralized with 1N HCl. DNA was precipitated with 20% TCA and trapped on G/C filter. The radioactivity was counted with a scintillation counter. The statistic analysis was performed by One factor ANOVA analysis.
